# IRF8 and its related molecules as potential diagnostic biomarkers or therapeutic candidates and immune cell infiltration characteristics in steroid-induced osteonecrosis of the femoral head

**DOI:** 10.1186/s13018-022-03381-1

**Published:** 2023-01-10

**Authors:** Xue-Zhen Liang, Xiao-Chen Liu, Song Li, Ming-Tao Wen, Yan-Rong Chen, Di Luo, Bo Xu, Nian-Hu Li, Gang Li

**Affiliations:** 1 Orthopaedic Microsurgery, Affiliated Hospital of Shandong University of Traditional Chinese Medicine, 16369 Jingshi Road, Jinan City, 250014 Shandong Province China; 2 The First Clinical Medical School, Shandong University of Traditional Chinese Medicine, Jinan, 250355 Shandong China; 3 Library, Shandong University of Traditional Chinese Medicine, Jinan, 250355 Shandong China; 4 Spinal Orthopedics, Affiliated Hospital of Shandong University of Traditional Chinese Medicine, 16369 Jingshi Road, Jinan City, 250014 Shandong Province China

**Keywords:** Steroid-induced osteonecrosis of the femoral head, Immune cell infiltration, Noncoding RNA, Competing endogenous RNA, Connectivity map database, Drug gene interaction database, L1000 fireworks display database, Molecular docking

## Abstract

**Purpose:**

Steroid-induced osteonecrosis of the femoral head (SONFH) was a refractory orthopedic hip joint disease in the young and middle-aged people, but the pathogenesis of SONFH remained unclear. We aimed to identify the potential genes and screen potential therapeutic compounds for SONFH.

**Methods:**

The microarray was obtained for blood tissue from the GEO database, and then it identifies differentially expressed genes (DEGs). The DEGs were analyzed to obtain the differences in immune cell infiltration. The gene functional enrichment analysis of SONFH was analyzed. The PPI of DEGs was identified through the STRING database, and the cluster modules and hub genes were ascertained using MCODE and CytoHubba, and the ROC curve of hub genes was analyzed, and the tissues distribution of hub genes was understood by the HPA, Bgee and BioGPS databases. The hub genes and target miRNAs and corresponding upstream lncRNAs were predicted by TargetScan, miRDB and ENCORI database. Subsequently, we used CMap, DGIdb and L1000FWD databases to identify several potential therapeutic molecular compounds for SONFH. Finally, the AutoDockTools Vina, PyMOL and Discovery Studio were employed for molecular docking analyses between compounds and hub genes.

**Results:**

The microarray dataset GSE123568 was obtained related to SONFH. There were 372 DEGs including 197 upregulated genes and 175 downregulated genes by adjusted *P* value < 0.01 and |log_2_FC|> 1. Several significant GSEA enrichment analysis and biological processes and KEGG pathway associated with SONFH were identified, which were significantly related to cytoskeleton organization, nucleobase-containing compound catabolic process, NOD-like receptor signaling pathway, MAPK signaling pathway, FoxO signaling pathway, neutrophil-mediated immunity, neutrophil degranulation and neutrophil activation involved in immune response. Activated T cells CD4 memory, B cells naïve, B cells memory, T cells CD8 and T cells gamma delta might be involved in the occurrence and development of SONFH. Three cluster modules were identified in the PPI network, and eleven hub genes including FPR2, LILRB2, MNDA, CCR1, IRF8, TYROBP, TLR1, HCK, TLR8, TLR2 and CCR2 were identified by Cytohubba, which were differed in bone marrow, adipose tissue and blood, and which had good diagnostic performance in SONFH. We identified IRF8 and 10 target miRNAs that was utilized including Targetsan, miRDB and ENCORI databases and 8 corresponding upstream lncRNAs that was revealed by ENCORI database. IRF8 was detected with consistent expression by qRT-PCR. Based on the CMap, DGIdb and L1000FWD databases, the 11 small molecular compounds that were most strongly therapeutic correlated with SONFH were estradiol, genistein, domperidone, lovastatin, myricetin, fenbufen, rosiglitazone, sirolimus, phenformin, vorinostat and vinblastine. All of 11 small molecules had good binding affinity with the IRF8 in molecular docking.

**Conclusion:**

The occurrence of SONFH was associated with a “multi-target” and “multi-pathway” pattern, especially related to immunity, and IRF8 and its noncoding RNA were closely related to the development of SONFH. The CMap, DGIdb and L1000FWD databases could be effectively used in a systematic manner to predict potential drugs for the prevention and treatment of SONFH. However, additional clinical and experimental research is warranted.

## Introduction

Osteonecrosis of the femoral head (ONFH) was a multifactorial refractory orthopedic hip joint disease in the young- and middle-aged people younger than 50 years, in which clinical manifestations appeared as the prolonged pain in the hip or groin area, occasional knee pain and limited hip rotation movement [1, 2]. In China, a national epidemiological survey of the general population showed that about 8.12 million cases were diagnosed as non-traumatic ONFH, and steroid-induced osteonecrosis of the femoral head (SONFH) accounted for the top incidences of non-traumatic ONFH [3]. Corticosteroid was well-known risk factor or associated condition for SONFH, but the mechanisms in the pathogenesis of SONFH were confusing and remained to be investigated [4]. The present study reported that SONFH was closely related to many theories in modern medicine, including the theory of fat embolism, increased intraosseous pressure, hypercoagulability state, steroid metabolism, immunity and the regulation of bone formation [5–7]. ONFH, including SONFH, was a progressive disease where 80% of these patients would collapse within 1–3 years without effective treatment [8]. Once the collapse of the femoral head appeared, the course of the disease would become difficult to reverse [5]. Early diagnosis of SONFH, even if non-surgical treatment alone, was effective in preventing disease progression and joint injury [9]. Male gender, longer symptom duration before treatment, higher visual analog acale and lower Harris hip score were negative prognostic factors after treatment for ONFH [10]. Drugs used for the prevention and treatment of SONFH included alendronate, enoxaparin, anticoagulants fibrinolysis promoters, vasodilators and traditional Chinese medicine (TCM), but the above drugs were mainly targeted to inhibit osteoclasts or increase osteogenesis and lacked specificity and directionality [10, 11]. Therefore, the identification of early diagnostic markers and suitable targeted molecular drugs would significantly benefit SONFH patients.

To data, an increasing number of studies had shown that immune cell infiltration played a significant role in the initiation, progression and metastasis of SONFH. CIBERSORT, an analysis tool that used high-throughput transcriptomic data to evaluate the expression of immune cells and obtained various immune cell proportions from samples, had been widely employed in a variety of diseases, such as osteoarthritis [12], lupus nephritis [13], atopic dermatitis [14] and cancer [15]. Recently, increasing evidence suggested that the lncRNAs could regulate mRNA expression indirectly via competitively binding a shared miRNA. The Encyclopedia of RNA Interactomes (ENCORI) was mainly focus on miRNA-target interactions including miRNA-ncRNA, miRNA-mRNA, ncRNA-RNA, RNA-RNA, RBP-ncRNA and RBP-mRNA interactions from multi-dimensional sequencing data [16]. Several lncRNAs acting as competing endogenous RNAs (ceRNAs) to regulate the target gene expression had been reported in cancers [17]. In recent years, L1000 Fireworks Display (L1000FWD), the Drug Gene Interaction Database (DGIdb) and the Connectivity Map (CMap) were used to predict existing small drugs by comparing identified differentially expressed genes (DEGs) of diseases [18, 19]. This strategy had successfully been applied for different types of diseases, such as cancer [20], muscle atrophy [21], acute myelogenous leukemia [22] and Parkinson’s disease [23]. To our knowledge, so far, no studies have used CIBERSORT, ENCORI, L1000FWD, DGIdb and CMap database to analyze immune cell infiltration, ceRNA network and potential targeted drugs in SONFH.

The National Center for Biotechnology Information (NCBI) Gene Expression Omnibus (GEO) database [7], a database for gene expression and hybridization array data, contained a wide assortment of high-throughput experimental data for various diseases, including SONFH. In this study, we first downloaded the high-throughput dataset of SONFH from the GEO database and screened the differential expression genes of SONFH. Subsequently, we used CIBERSORT to analyze the differential expression genes in immune infiltration between SONFH and normal samples in 22 immune cell subsets. In addition, we used CytoHubba to identify hub genes and used ENCORI to perform the ceRNA network with the hub genes. Finally, we used L1000FWD, DGIdb and CMap databases to screen potential candidate drugs, which might provide a novel field in understanding the pathological mechanism and therapeutic concept of SONFH.

## Method

### Microarray data quality assessment and identification of DEGs

The microarray dataset was used to obtained for blood tissue from NCBI GEO (https://www.ncbi.nlm.nih.gov/geo/) database in SONFH and non-SONFH. The following keywords were used as a screening criterion: (1) “Steroid induced Osteonecrosis of the Femoral Head” or “SONFH”; (2) *Homo sapiens*; and (3) “Expression Profiling by array” or “Expression profiling by high throughput sequencing.” Finally, only one datasets GSE123568 (https://www.ncbi.nlm.nih.gov/geo/query/acc.cgi?acc=GSE123568), which was submitted on Dec 10, 2018 and updated on Jan 01, 2020 by Zhang et al. and included 10 non-SONFH (following steroid administration) samples and 30 SONFH samples. The platform of the dataset was GPL15207 [PrimeView] Affymetrix Human Gene Expression Array. The raw data of GSE123568 were downloaded as CEL files, and the platform files were downloaded as TXT files.

We used the robust multiarray average (RMA) method based on the “affy” R package to pre-process and normalize the original files. The high-dimensional sequencing data were projected into two-dimensional space for principal component analysis (PCA) to observe the overall distribution among groups of samples and identify the presence of singular samples. We then computed and visualized the PCA using “PCA” and “ggplot2” R package.

We used “limma” R package to analysis the DEGs. The screening criteria were |log_2_ (fold change)|> 1 and adjusted *P* value < 0.01. To better visualize the DEGs, the heatmaps and volcano plots are used by the “pheatmap” and “ggplot2” R package.

### Evaluation of immune cell infiltration

The gene expression matrix data of GSE123568 were uploaded to CIBERSORT database to obtain the immune cell infiltration matrix. The permutation was set as 1000, and the cutoff criterion was set as *P* < 0.05. Then the “ggplot2” R package was used to perform PCA clustering analysis on immune cell infiltration matrix data to draw a two-dimensional PCA map. The “corrplot” R package was used to draw a correlation heatmap to visualize the correlation of 22 types of infiltrating immune cells, and the “ggplot2” package was used to draw violin diagrams to visualize the differences in immune cell infiltration [24].

### Functional enrichment analysis

Gene set enrichment analysis (GSEA, http://www.broadinstitute.org/gsea/index.jsp) was a computational method that was normally used to analyze the distribution of the predefined genes in ranked genes associated with complex phenotypes, which was developed by the Broad Institute website. The biological pathways with normalized enrichment score (NES) larger than 0 presented the biological pathways were activated, while NES smaller than 0 presented the biological pathways was suppressed. To investigate the potential mechanisms underlying the progression of SONFH, GSEA was conducted to screen out whether some biological pathways showed statistically significant differences between non-SONFH and SONFH groups according to the MSigDB molecular signatures database (version 7) and c2.cp.v7.2.symbols.gmt [Curated] gene sets with the “GSEA” and “clusterProfiler” R package. We set the parameter of require fields as follows: expression dataset as no collapse, number of permutations as 1000 and permutation type as phenotype. The significance threshold was set at a false discovery rate (FDR) < 0.25 and adjusted *P* value < 0.05.

In addition, to analyze the identified DEGs, the gene ontology (GO) and Kyoto Encyclopedia of Genes and Genomes (KEGG) pathway enrichment analysis were performed and visualized using the “clusterProfiler” and “GOplot” R package. The criterion for significantly enriched terms was set as adjusted *P* value < 0.05. The GO enrichment analyses mainly were divided into the biological process (BP), cellular component (CC) and molecular function (MF), whereas KEEG enrichment analyses demonstrated the significance of potential pathways associated with the DEGs.

### Construction of protein–protein interaction (PPI) network and screening of hub genes and key modules

To gain more insight into the in-depth relationships among the DEGs, we predicted and constructed the PPI network of DEGs using the Search Tool for the Retrieval of Interacting Genes (STRING, version: 11.0, https://string-db.org/). The cutoff criterion of a combined score was set to medium confidence ≥ 0.4, and the isolated nodes were discarded. Immediately after, the PPI network was constructed and visualized using the Cytoscape software (version: 3.7.2, http://cyto-scape.org/).

We utilized CytoHubba, a plug-in of Cytoscape, to screen hub genes with the nodes found by the three algorithms including degree, maximal clique centrality (MCC) and maximum neighborhood component (MNC). The final hub genes were identified as the intersection of top 20 hub genes calculated by the each algorithm.

Concurrently, we analyzed the each clustered using molecular complex detection (MCODE), a plug-in of Cytoscape. The higher the module scores, the more the number of close interactions and enrichment it played, and the cutoff criterion was set as the k-core ≥ 5. Furthermore, the GO and KEGG pathway enrichment analyses of DEGs in the most significantly clustered modules were carried out by the “clusterProfiler” R package.

### ROC curve of hub genes

The final specifically expressed hub genes expression profiles of non-SONFH and SONFH samples were analyzed, and the ROC curve was drawn using “pROC” R package. The area under the curve (AUC) could represent the intrinsic effectiveness of diagnostic tests, which was an indicator combining sensitivity and specificity between 0.5 and 1. The closer the AUC was to 1, the better the diagnosis was. The AUC had low accuracy at 0.5–0.7, moderate accuracy at 0.7–0.9 and high accuracy above 0.9.

### Identification of the tissues

To understand the specific expression of these hub genes, the Human Protein Atlas project (HPA, http://www.proteinatlas.org/), Bgee (https://bgee.org/) and BioGPS (http://biogps.org/#goto=welcome) databases were used to analyze the tissue distribution in human bone marrow, adipose tissues and blood. The purpose of the HPA database was to map all the human proteins in cells, tissues and organs using an integration of various omics technologies, including antibody-based imaging, mass spectrometry-based proteomics, transcriptomics and systems biology, which was funded by the Knut & Alice Wallenberg foundation. The Bgee was a database for retrieval and comparison of gene expression patterns across multiple animal species, which provided an intuitive answer to the question "where is a gene expressed," and supported research in cancer and agriculture as well as evolutionary biology. The BioGPS database was a free extensible and customizable gene annotation portal and a complete resource for learning about gene and protein function.

### Prediction of target miRNAs and construction of the mRNA-miRNA network

The three online miRNA databases, including TargetScan (http://www.targetscan.org/vert_72/), miRDB (http://mirdb.org/) and ENCORI (version 3.0, http://starbase.sysu.edu.cn/index.php), were used to predict target miRNAs of hub genes, and the final miRNAs were selected at least three databases. Next, we used Cytoscape software to construct the mRNA-miRNA network based on the relationship between miRNA and target mRNAs.

### Prediction of target corresponding upstream lncRNAs and construction of ceRNA networks

The regulatory mechanism constituted the basis of ceRNA interplay networks, which the upstream molecules as miRNA sponges, including lncRNA, circRNA and pseudogene, could regulate the function of miRNA in the regulation of mRNAs by combining miRNA response elements (MREs). We used ENCORI to predict lncRNAs by the tools of mRNA-ceRNA network. The parameter of the mRNA-ceRNA network was set as the followed screening criteria: mammalian, human genome, h19 assembly, miRNA number ≥ 2, *P* value ≤ 0.01, FDR ≤ 0.01, and with or without Pan-Cancer data. And then, we also used ENCORI to identify the intersections among the predicted lncRNAs based on mRNA-ceRNA network, hub genes and target miRNAs. Finally, ceRNA networks based on above regulatory relationships were constructed, visualized and analyzed by Cytoscape software.

### Cross-validation of the external dataset and quantitative real-time-PCR (qRT-PCR) for the hub genes in SONFH

A total of 14 cartilage samples (including 7 SONFH and 7 control samples with healthy state) were collected from Affiliated Hospital of Shandong University of Traditional Chinese Medicine. The Ethical Committee of Affiliated Hospital of Shandong University of Traditional Chinese Medicine approved this study, and the respective patient provided informed consent in a written form. At artificial hip replacement, samples of femoral neck fracture and SONFH were collected, the cartilage of 1*1 cm^2^ was immediately frozen in liquid nitrogen, and RNA was extracted according to the protocol. The mRNA transcripts were quantified by qRT-PCR using Thermo Scientific™ NanoDrop Lite and a CFX96 Touch Real-Time PCR Detection System (BIO-RAD, CFX96, USA).

The amplification conditions were set as follows: 95 °C for 10 min, followed by 40 cycles of 95 °C for 15 s, 60 °C for 30 s and 60 °C for 30 s. Then, a melting curve was established to obtain the experimental data. GAPDH was used as the reference gene, and all qRT-PCRs were conducted in triplicates. A standard comparative method (ΔΔCt) was used to evaluate the expression stability of the potential candidate genes. Relative target gene expression levels were calculated using the 2^−ΔΔCt^ method. The sequences of primers used in this study are listed in Table 1.

**Table 1:** Lists of primer sequences, amplicon length used for qRT-PCR

Gene symbol	Forward primer (5′–3′)	Reverse primer (5′–3′)	Amplicon length (bp)	Annealing temperature (°C)
GAPDH	GTGAGATCGGTAGGTTGGTG	CCTTGACTTTGAGCTCGTGA	159	60
IRF8	GATCCCTTGGAAACACGCTG	GCCACGCCTAGTTTGCATTT	264	60

#### Identification of the potential drugs

The potentially therapeutic drugs for SONFH were predicted using the L1000FWD, DGIdb and CMap, and the final potential drugs were selected at least three databases.

The L1000FWD database recorded interactive visualization of gene expression signatures of upregulated and downregulated genes induced over 16,000 drugs and small molecules in cancer cell lines. If the lists of genes we uploaded coincided the ones stored in the database, then the drugs or small molecules mapping the lists of these genes considered similar; if not consistent, it was thought to be opposite. Therefore, we could predict small molecules by comparing identified DEGs between non-SONFH and SONFH samples, and the cutoff criterion for screening drugs and small molecules was set as similarity score < − 0.03.

The DGIdb database was a web-based data visualization application that revealed various types of information related to more than 40,000 genes and 10,000 drugs, which recorded the information on interactions between drugs and their candidate gene lists. Using these sources of information in the database, we could upload the identified DEGs between non-SONFH and SONFH samples to identify their interacting drugs.

The CMap database, a pioneering database of chemically induced transcriptome data on human cell lines, recorded 7000 drug-associated gene expression profiles of five cancer cell lines perturbed by 1309 compounds, which could identify a large collection of drugs and small molecule compounds that had potential therapeutic effects. The drugs with significantly negative scores that was close to − 1 were predicted as therapeutic medications for disease in the database. So, we performed an in silico drug screening for SONFH using CMap by uploading Affymetrix U133A probe IDs of upregulated and downregulated genes, and the cutoff criterion used to identify potential drug candidates was set as the CMap score ≤ − 0.65 [25].

### Molecular docking between small molecular compounds and the hub genes

The hub genes and small molecular compounds were selected for the molecular docking analysis. The sdf structure of potentially therapeutic relevant small molecular compounds was downloaded from the pubChem database (https://pubchem.ncbi.nlm.nih.gov/) [18] and saved in the pdbqt format as an active small molecule in molecular docking by Chemoffice software. The 3D protein structures of the hub genes were downloaded from the RCSB Protein Data Bank (PDB) database (https://www.rcsb.org/) [19]. The PyMOL 2.5.2 software was used to perform operations such as removing solvent and removing organic, and the file was then saved in the PDB format. The AutoDockTools software was used for adding hydrogen atoms, calculating Gasteiger-Marsili charges, adding partial charges and generating PDBQT files. The AutoDockTools Vina 1.5.6 software was used for semi-flexible docking, the docking results for the highest scoring conformation were visualized and analyzed using PyMOL 2.5.2 and Discovery Studio 2017 software. If the binding free energy required for the interaction between the ligand and the receptor was less than 0 kcal mol^−1^, it indicated that the two could bind spontaneously. If the binding free energy was less than − 5 kcal mol^−1^, it indicated that the two had good binding affinity. The binding energy ≤ − 5.0 kcal mol^−1^ suggested a better affinity.

### Statistical analysis

Data were presented as means (± standard deviation) or counts (percentages), and the statistical analysis of the data was performed with R software (version 4.0.2) and GraphPad Prism 9 in this study. The gene expression levels of samples in the GSE123568 and GSE74089 samples were compared using the Kruskal–Wallis test. *P* < 0.05 was set as the threshold for significant statistical significance.

## Results

### Microarray data processing and identification of DEGs

**Fig. 1:**
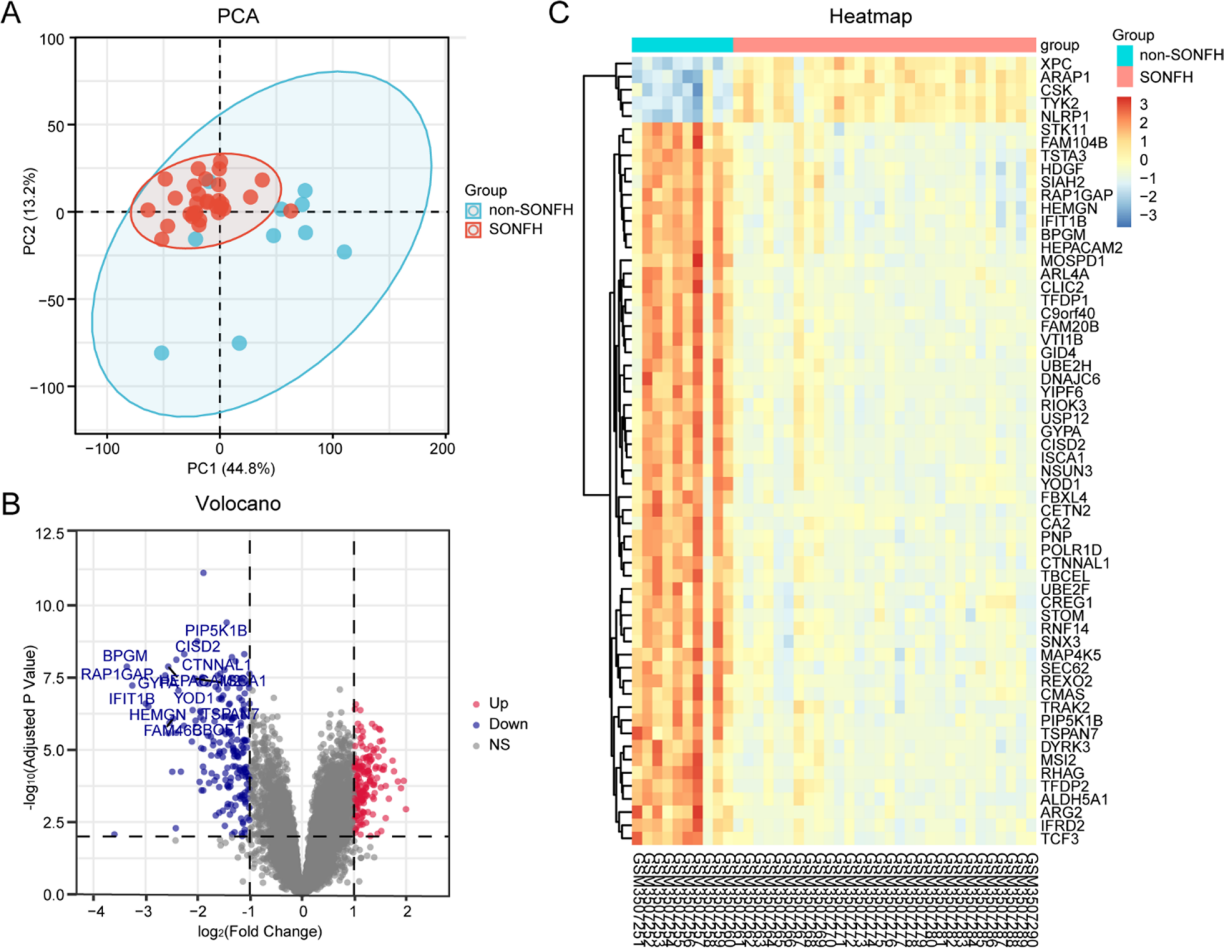
Microarray data processing and identification of DEGs of GSE123568 in SONFH samples compared to non-SONFH samples. **A** PCA of GSE123568 in SONFH samples compared to non-SONFH samples. The blue dot represented non-SONFH samples, and the red dot represented SONFH samples. **B** Volcano plots of DEGs. Data points in red represent upregulated, and blue represent downregulated genes. The differences were set as |log_2_FC|> 1 and adjusted P value < 0.01. The text was added directly to the plot by adjusted *P* value < 0.000001 and |log_2_FC|> 2. **C** Hierarchical clustering heatmap of the top 60 DEGs sorted by adjusted *P* value. Legend on the top right indicates the log_2_FC

The microarray dataset GSE123568 about identification of potential biomarkers for improving the precision of early detection of SONFH were downloaded from GEO database, which included the 30 SONFH patients and 10 non-SONFH patients (following steroid administration). In the aspect of overall design of GSE123568, the gene expression profiles were detected by microarray analysis based on SONFH patients and non-SONFH patients. Then, a list of candidate gene biomarkers of SONFH were identified by integrating differential expression data analysis and gene signal transduction network analysis. Considering that the quality of the samples was critical for subsequent analysis, the original CEL files were used to evaluate the quality of the microarray while standardizing the data. We next subjected above data to principal components analysis (PCA), which confirmed the cluster analysis data and reduced the number of input variables for the subsequent advanced statistical analysis. Samples from non-SONFH patients were clustering together and maintained a significant distance from the SONFH patients shown in Fig. 1A. Overall, the quality of the microarray dataset was considered reliable.

**Fig. 2:**
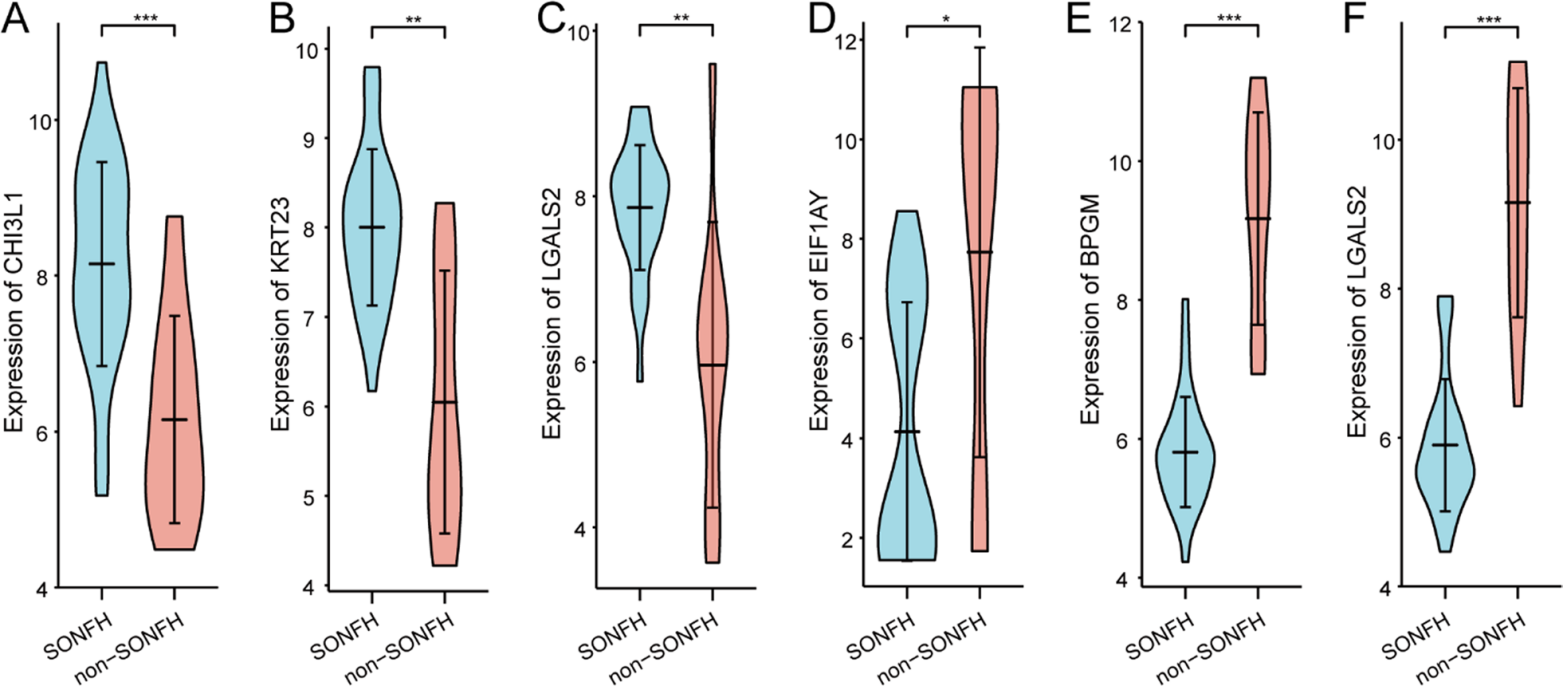
Violin plot of gene expressions for the top 3 upregulated and downregulated mRNAs based on fold change

There were 372 DEGs extracted from GSE123568 based on the defined criteria of an adjusted *P* value < 0.01 and |log_2_FC|> 1, which included 175 upregulated genes and 197 downregulated genes in SONFH samples compared to non-SONFH samples. The DEGs were shown in the volcano plots and the hierarchical clustering heatmap by the “ggplot” and “pheatmap” R package in Fig. 1B, C, and the top 3 upregulated and downregulated mRNAs are shown in violin plot in Fig. 2.

### Enrichment analysis

**Fig. 3:**
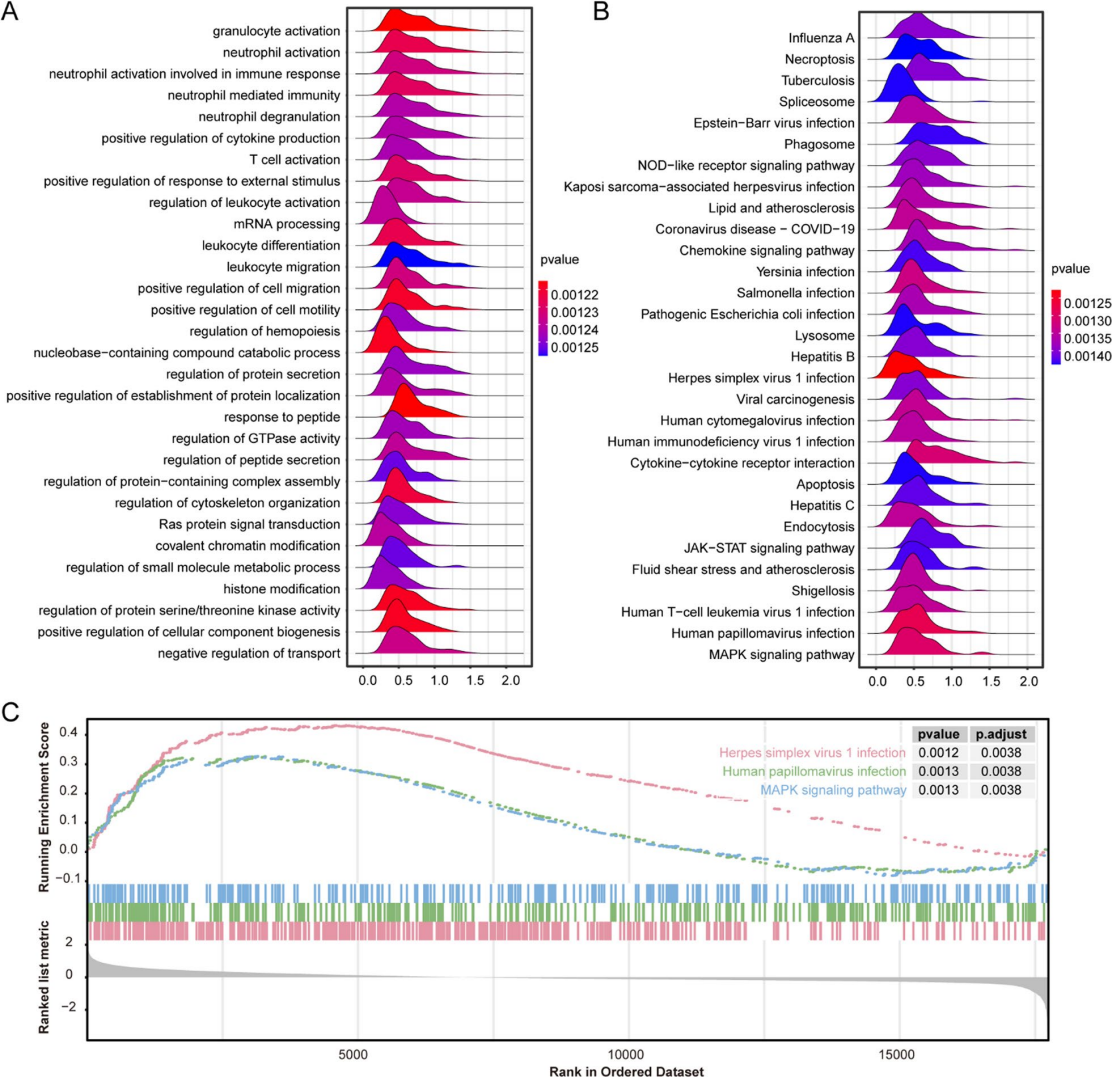
GSEA analysis of the most enriched gene sets of all detected genes in the non-SONFH and SONFH samples in the GSE123568. **A** The GSEA analysis ridgeplot of top 30 significant-enriched GO pathway gene sets. **B** The GSEA analysis ridgeplot of top 30 significant-enriched KEGG pathway gene sets. **C**The GSEA plot of top 3 most significant upregulated enriched KEGG pathway gene sets

The GSEA enrichment analysis was used to assess the distribution trend of a predefined set of genes in lists of degree sequencing associated with the phenotype, thus judging its contribution to the phenotype. In order to unravel whether the identified sets of genes showed statistical differences between non-SONFH and SONFH patients for the abovementioned data, GSEA was analyzed and visualized based on the c2 gene sets in the MSigDB database using “GSEA” and “clusterProfiler” R package in Fig. 3.

As shown in Table 2, the GO biological process terms, such as regulation of cytoskeleton organization (NES = 1.594, *P* value = 0.001), nucleobase-containing compound catabolic process (NES = 1.733, *P* value = 0.001), positive regulation of cellular component biogenesis (NES = 1.530, *P* value = 0.001) and leukocyte differentiation (NES = 1.941, *P* value = 0.001) were significantly enriched in SONFH patients.

**Table 2:** The GSEA analysis of top 10 significant-enriched GO (BP, CC and MF)gene sets

ONTOLOGY	ID	Description	Set size	Enrichment score	NES	*P* value
BP	GO:0051493	Regulation of cytoskeleton organization	495	0.331	1.594	0.001
BP	GO:0034655	Nucleobase-containing compound catabolic process	499	0.360	1.733	0.001
BP	GO:0044089	Positive regulation of cellular component biogenesis	499	0.318	1.530	0.001
BP	GO:0002521	Leukocyte differentiation	496	0.403	1.941	0.001
BP	GO:2000147	Positive regulation of cell motility	492	0.372	1.787	0.001
BP	GO:0036230	Granulocyte activation	485	0.532	2.551	0.001
BP	GO:1901652	Response to peptide	485	0.345	1.654	0.001
BP	GO:0032103	Positive regulation of response to external stimulus	474	0.462	2.214	0.001
BP	GO:0071900	Regulation of protein serine/threonine kinase activity	483	0.322	1.543	0.001
BP	GO:0042119	Neutrophil activation	480	0.531	2.543	0.001
CC	GO:0098552	Side of membrane	493	0.389	1.869	0.001
CC	GO:0010008	Endosome membrane	452	0.425	2.024	0.001
CC	GO:0005635	Nuclear envelope	435	0.352	1.678	0.001
CC	GO:0000785	Chromatin	454	0.316	1.504	0.001
CC	GO:0030055	Cell-substrate junction	406	0.395	1.873	0.001
CC	GO:0005925	Focal adhesion	400	0.400	1.895	0.001
CC	GO:0005774	Vacuolar membrane	386	0.434	2.053	0.001
CC	GO:0031252	Cell leading edge	389	0.354	1.677	0.001
CC	GO:0030133	Transport vesicle	369	0.309	1.458	0.001
CC	GO:0031300	intrinsic component of organelle membrane	351	0.331	1.553	0.001
MF	GO:0008047	Enzyme activator activity	473	0.326	1.562	0.001
MF	GO:0050839	Cell adhesion molecule binding	460	0.327	1.559	0.001
MF	GO:0031267	Small GTPase binding	413	0.334	1.582	0.001
MF	GO:0017016	Ras GTPase binding	399	0.330	1.565	0.001
MF	GO:0005543	Phospholipid binding	401	0.311	1.471	0.001
MF	GO:0042578	Phosphoric ester hydrolase activity	353	0.341	1.598	0.001
MF	GO:0033218	Amide binding	322	0.360	1.673	0.001
MF	GO:0045296	Cadherin binding	306	0.343	1.585	0.001
MF	GO:0140098	Catalytic activity, acting on RNA	335	0.376	1.748	0.001
MF	GO:0060589	Nucleoside-triphosphatase regulator activity	312	0.338	1.562	0.001

We found that 54 KEGG pathway gene sets were significantly enriched at FDR (*q*-value) < 0.25 and adjusted *P* value < 0.05, which included 51 gene sets positively correlated with the SONFH patients, such as NOD-like receptor signaling pathway, MAPK signaling pathway, FoxO signaling pathway and insulin signaling pathway, and which included 3 gene sets positively correlated with the SONFH patients, including cell cycle, biosynthesis of cofactors and olfactory transduction.

**Fig. 4:**
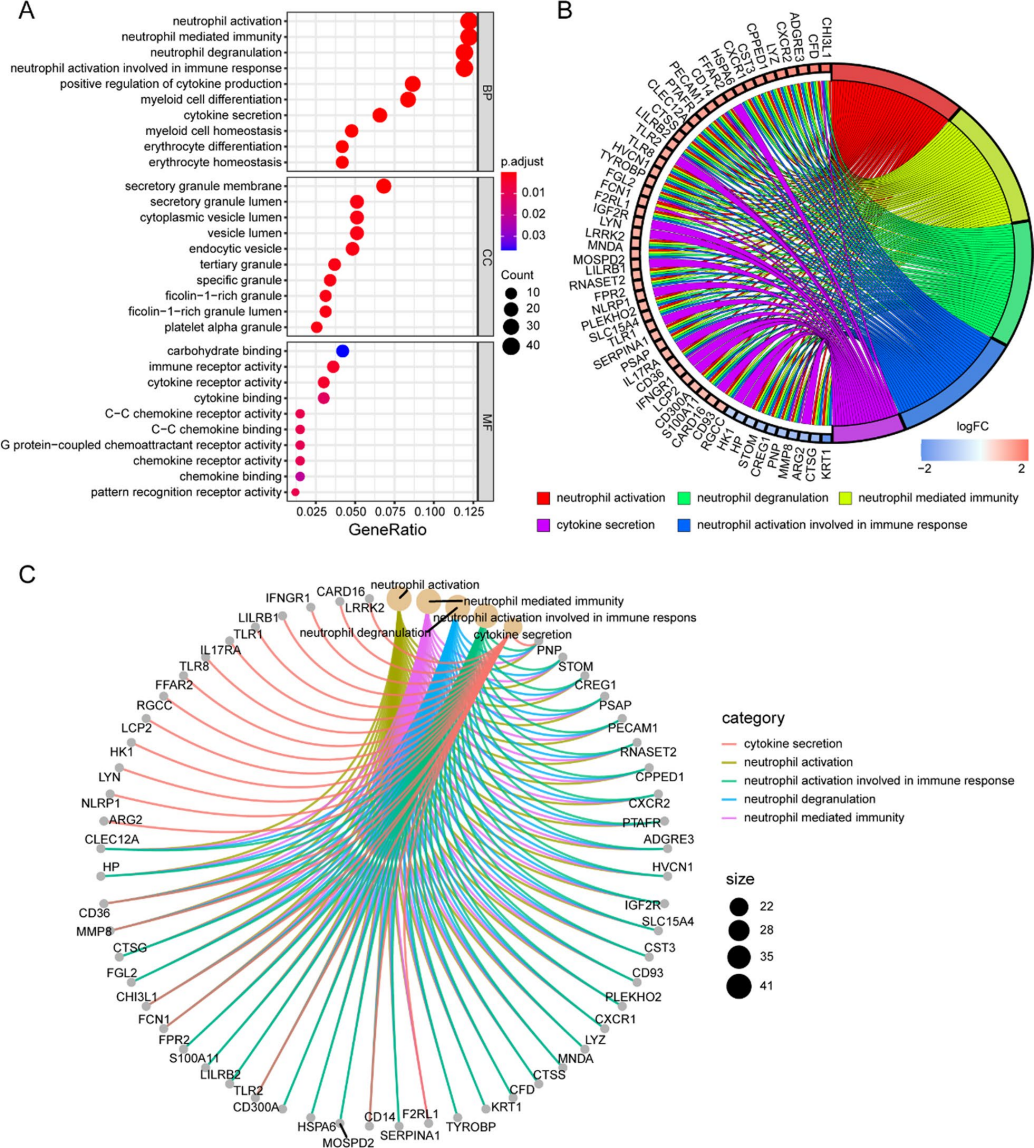
Gene ontology (GO) enrichment analysis of DEGs of GSE123568. **A** Advanced bubble chart showed the top 10 GO enrichment significance items of DEGs sorted by adjusted P value in BP, CC and MF. The x-axis label represented the gene ratio, and the y-axis label represented GO terms. **B** Chord plot showed the distribution of DEGs in different GO-enriched functions. Symbols of DEGs were presented on the left side of the graph with their fold change values mapped by color scale. Gene involvement in the GO terms was determined by colored connecting lines. **C** Cnet plot showed the relationship between the DEGs and GO terms

**Fig. 5:**
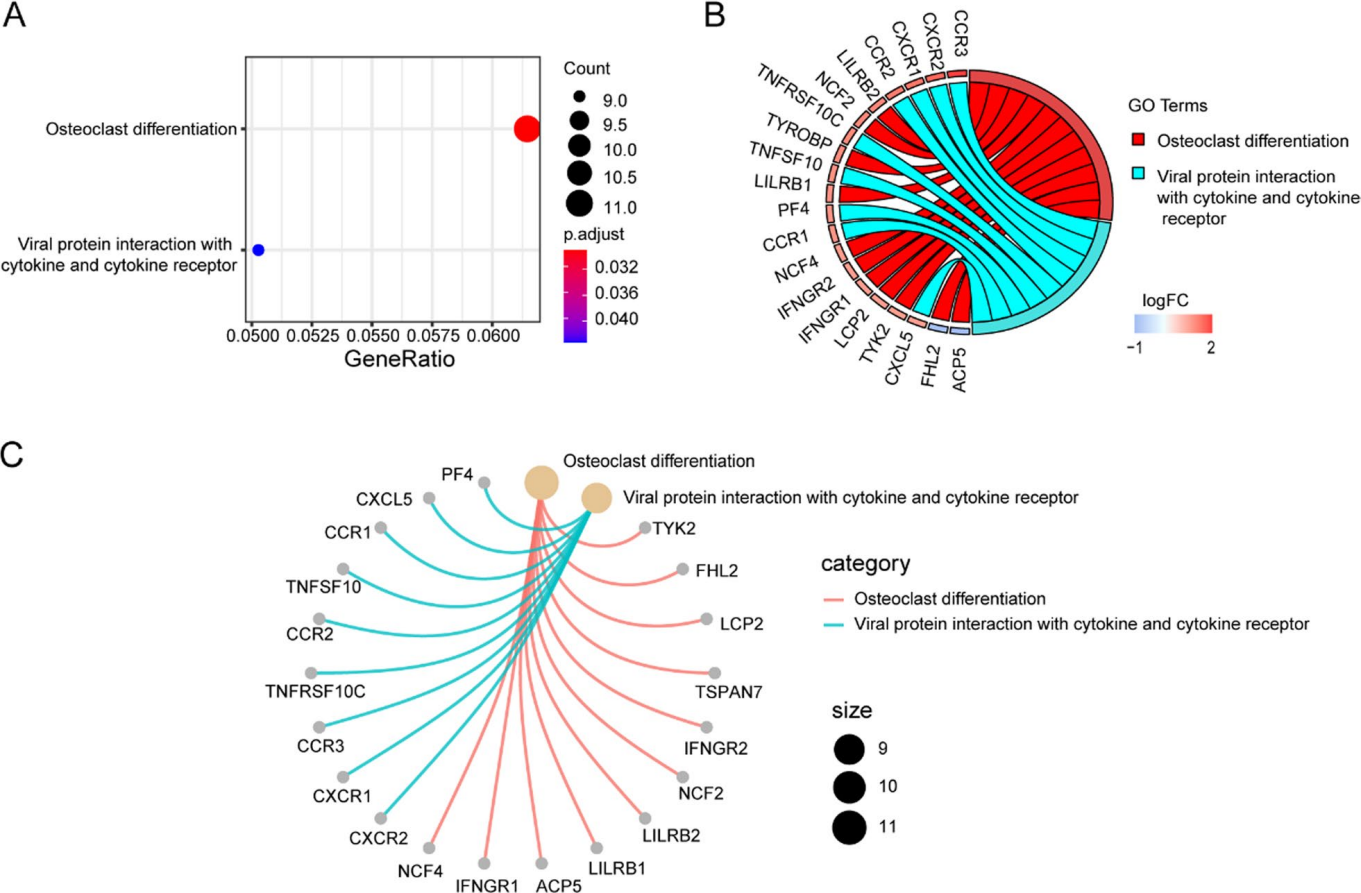
KEGG pathway analysis of DEGs of GSE123568. **A** Advanced bubble chart showed enrichment of DEGs in signaling pathways. The x-axis label represented the gene ratio and the y-axis label represented pathway. **B** Chord plot showed the distribution of DEGs in different KEGG pathways. Symbols of DEGs were presented on the left side of the graph with their fold change values mapped by color scale. Gene involvement in the KEGG pathways was determined by colored connecting lines. **C** Cnet plot showed the relationship between the DEGs and KEGG pathways

Next, to uncover molecular mechanisms involved of 372 DEGs in the GSE123568, the enriched GO and KEGG pathway analysis were performed and visualized in “clusterProfiler,” “Goplot” and “pathview” R package in Figs. 4 and 5.

The result of GO enrichment indicated that for BP, the DEGs were significantly enriched in neutrophil activation, neutrophil-mediated immunity, neutrophil degranulation, neutrophil activation involved in immune response, cytokine secretion, myeloid cell differentiation, positive regulation of cytokine production and so on. Regarding CC, the DEGs were significantly enriched in the secretory granule membrane, tertiary granule, ficolin-1-rich granule, ficolin-1-rich granule lumen, secretory granule lumen, cytoplasmic vesicle lumen, vesicle lumen, specific granule, endocytic vesicle, platelet alpha granule, and so on. For MF, the DEGs were significantly enriched in immune receptor activity, cytokine receptor activity, pattern recognition receptor activity, C–C chemokine receptor activity and C–C chemokine binding.

The result of KEGG pathway enrichment analysis indicated that the DEGs were mainly enriched in osteoclast differentiation and viral protein interaction with cytokine and cytokine receptor and so on.

Collectively, these results further verified that the gene transcripts expressed significantly differently between non-SONFH and SONFH groups might be closely associated with osteoclast differentiation, apoptosis, cell cycle and immune cell infiltration.

### Immune cell infiltration

**Fig. 6:**
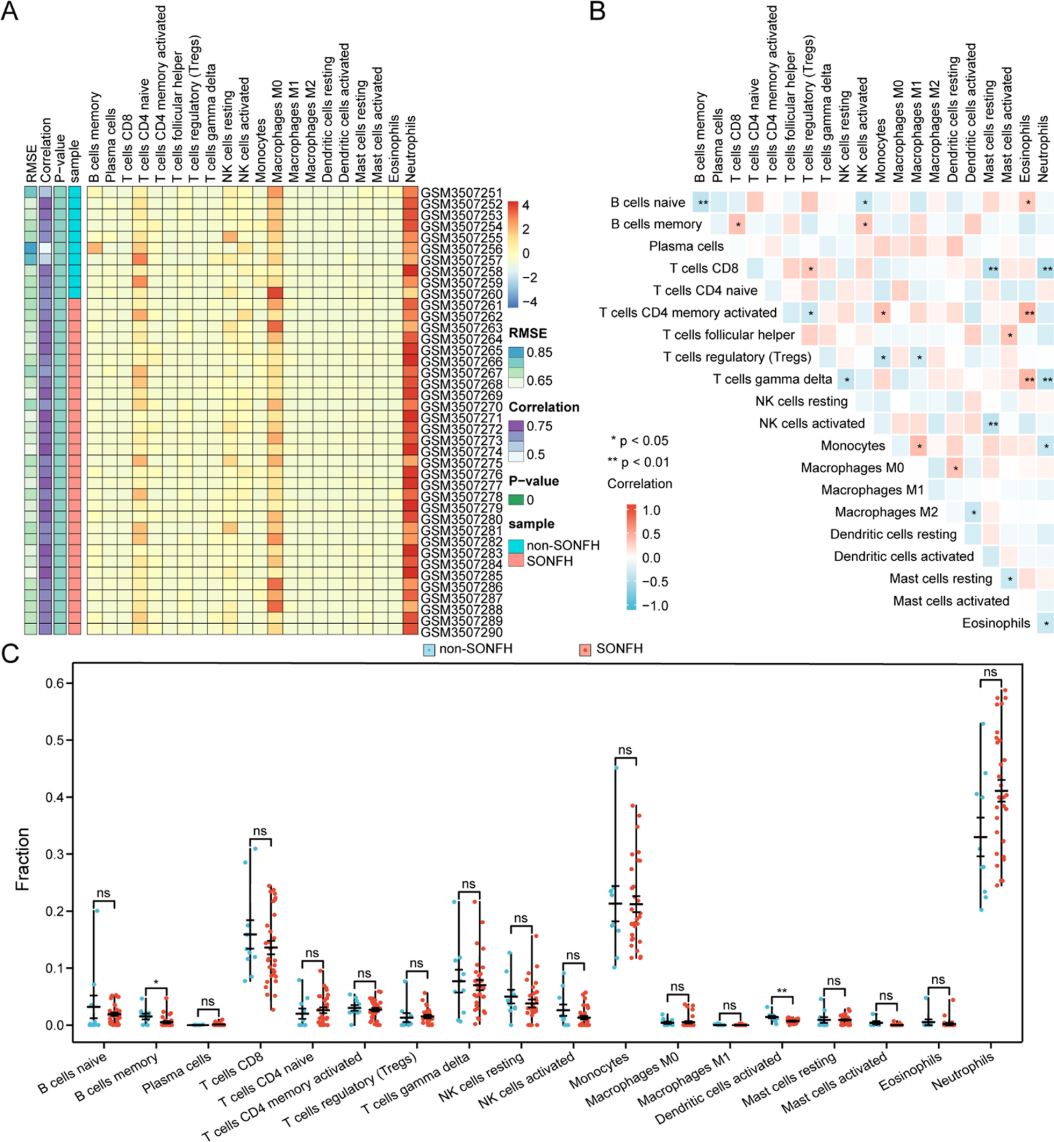
Evaluation and visualization of immune cell infiltration. **A** Heatmap plot of immune cell infiltration between non-SONFH samples and SONFH samples. **B** Spearman correlation analysis among 22 types of immune cells. The size of the colored squares represents the strength of the correlation; blue represent a positive correlation, red represents a negative correlation. The darker the color the stronger the correlation. * represents *P* < 0.05, ** represents *P* < 0.01. **C** Violin diagram of the proportion of 22 types of immune cells. The red marks represent the difference in infiltration between the two groups of samples

The PCA cluster analysis could be used to test the consistency of biological repetition and the difference of non-SONFH and SONFH groups. The heatmap analysis results of immune cell infiltration showed that there was a significant difference in immune cell infiltration between the SONFH sample and non-SONFH sample, which was shown in Fig. 6A. Correlation heatmap of the 22 types of immune cells revealed that activated T cells CD4 memory and T cells gamma delta had a significant positive correlation with eosinophils and that B cells naive had a significant negative correlation with B cells memory and that T cells CD8 had a significant negative correlation with mast cells resting and neutrophils and that T cells gamma delta had a significant negative correlation with Neutrophils and that activated NK cells had a negative correlation with Mast cells resting shown in Fig. 6B. The violin plot of the immune cell infiltration difference in SONFH sample showed that compared with the non-SONFH sample, B cells memory and activated dendritic cells infiltrated less are shown in Fig. 6C.

### Construction of PPI network, analysis of MCODE cluster modules and identification of hub genes

**Fig. 7:**
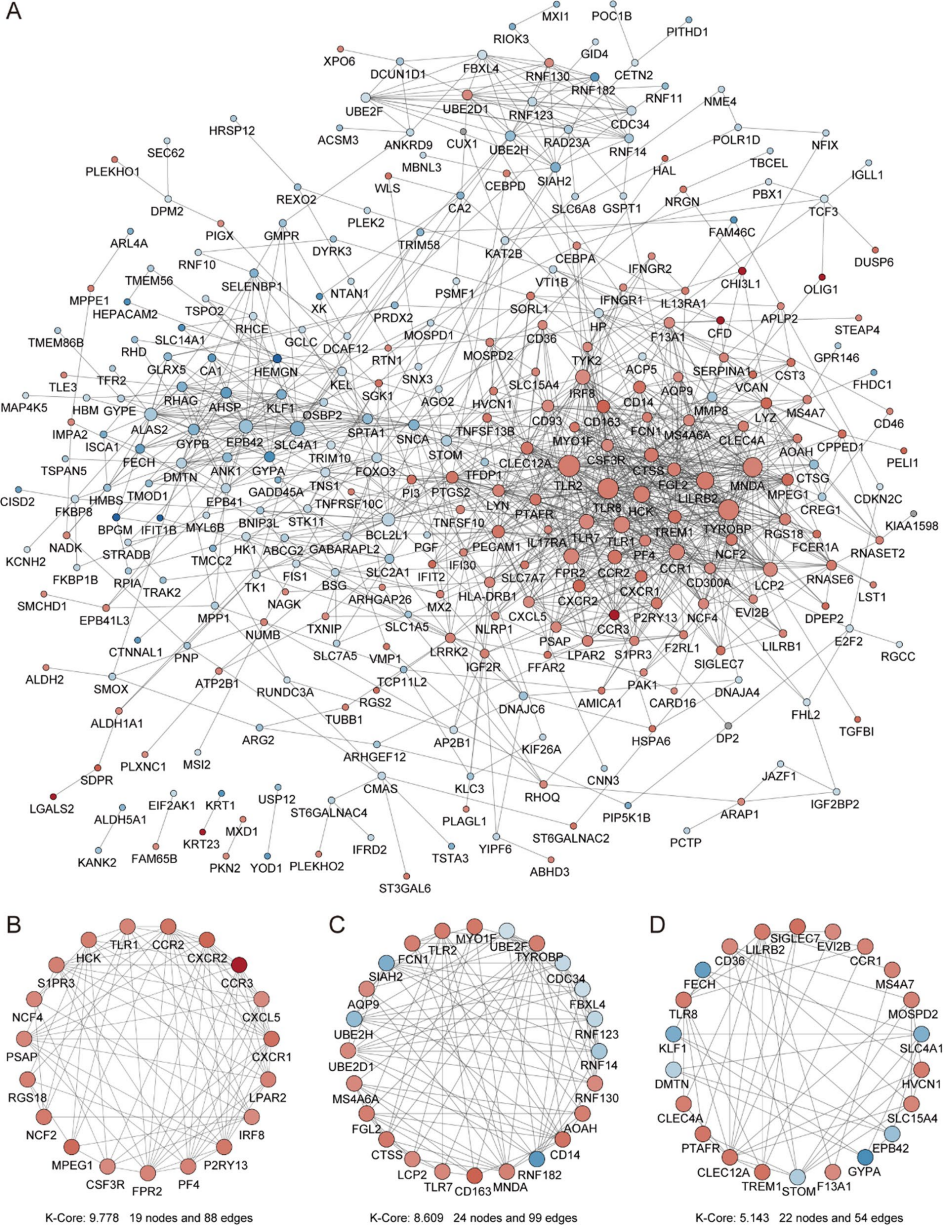
PPI network of DEGs and three cluster modules identified by MCODE. **A** The PPI network of DEGs was consisted of 300 nodes and 1192 edges. Each node represented one protein, while each edge represented one interaction. The color of nodes reflected the fold change, red color represented a upregulated gene, and blue color represented a downregulated gene, the darker the color indicated the greater the multiple of the fold change; the size of nodes reflected the degree value, the larger the node, the greater the degree value. **B** cluster 1: score 9.778, 19 nodes and 88 edges; **C** cluster 2: score 8.609, 24 nodes and 99 edges; **D** cluster 3: score 5.143, 22 nodes and 54 edges

The PPI network was consisted of 300 nodes and 1192 edges, which was based on STRING and visualized by Cytoscape in Fig. 7A.

The MCODE plug-in was applied to identify the significant gene cluster modules. Three cluster modules were identified in the network according to the filter criteria with degree cutoff = 2, node score cutoff = 0.2, k-core = 5 and max depth = 100, which visualized in Fig. 7B–D. Cluster 1 had the highest cluster score that was comprised of 19 nodes and 88 edges, which were all upregulated genes (score: 9.778), followed by cluster 2 that was comprised of 24 nodes and 99 edges (score: 8.609), cluster 3 that was comprised of 22 nodes and 54 edges (score: 5.143).

Immediately after, the CytoHubba plug-in was used to identify hub genes, and 11 hub genes were identified by intersecting the results within at least three algorithms from the four algorithms of Cytohubba including MCC, MNC, DMNC and degree, which are shown in Table 3. These hub genes were considered to be the most important genes in PPI network and might play a workhorse role in the pathogenesis of SONFH. Additionally, GO and KEGG enrichment analysis of the hub genes exhibited that the hub genes were mainly enriched in inflammatory and osteoclast differentiation by “clusterProfiler” and “Goplot” R packages. As the most common bone metabolic disease, better understanding of the inflammatory and osteoclast differentiation was an important part of current research in SONFH. The discovery of the hub genes in serum of SONFH patients might contribute to the discovery of key targets and establishment of hallmarks in the pathogenesis of SONFH.

**Table 3:** 11 hub genes identified by at least three algorithms of CytoHubba

Gene symbol	Gene description	Degree	log_2_FC	Algorithms
FPR2	Formyl peptide receptor 2	9	1.121	Degree, MNC, MCC
LILRB2	Leukocyte immunoglobulin like receptor B2	11	1.247	Degree, MNC, MCC
MNDA	Myeloid cell nuclear differentiation antigen	9	1.149	Degree, MNC, MCC
CCR1	C–C motif chemokine receptor 1	9	1.123	Degree, MNC, MCC
IRF8	Interferon regulatory factor 8	9	1.006	Degree, MNC, MCC
TYROBP	TYRO protein tyrosine kinase binding protein	12	1.202	Degree, MNC, MCC
TLR1	Toll like receptor 1	11	1.099	Degree, MNC, MCC
HCK	HCK proto-oncogene, Src family tyrosine kinase	12	1.204	Degree, MNC, MCC
TLR8	Toll like receptor 8	12	1.231	Degree, MNC, MCC
TLR2	Toll like receptor 2	13	1.239	Degree, MNC, MCC
CCR2	C–C motif chemokine receptor 2	9	1.357	Degree, MNC, MCC

### ROC curve of the 11 specifically targeted expressed hub genes in non-SONFH samples and SONFH samples

**Fig. 8:**
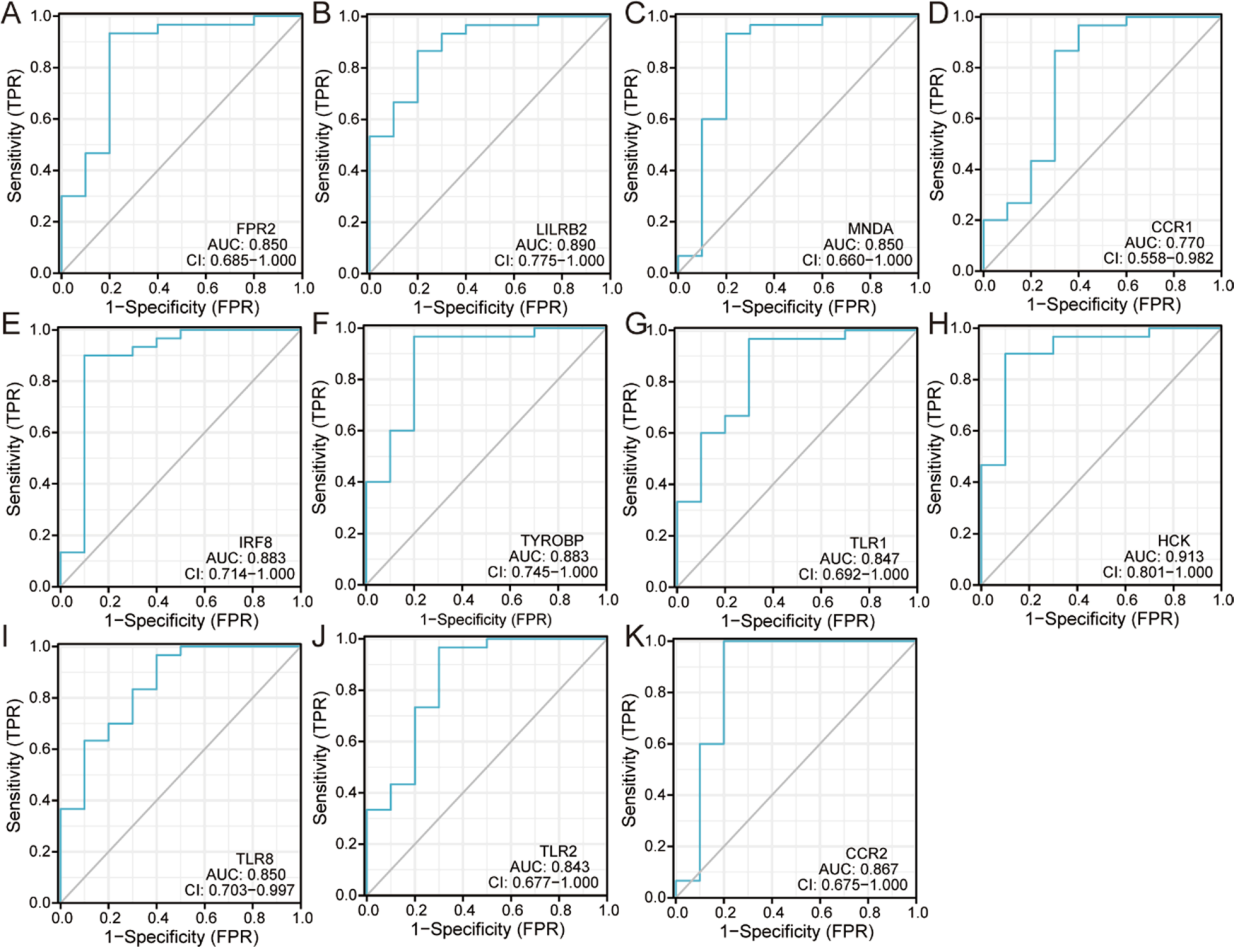
ROC curve of the 11 specifically expressed hub genes in SONFH samples

The ROC curves of 11 hub genes of SONFH samples were analyzed, established and drawn by “pROC” and “ggplot2” R package in Fig. 8. Area under the curve (AUC) values indicated an indicator combining sensitivity and specificity metrics, which could describe the intrinsic effectiveness of diagnostic tests. Compared to non-SONFH samples, these 11 hub genes had higher diagnostic value in the SONFH samples. Among them, HCK had the highest diagnostic value (AUC: 0.913) in the SONFH samples. The diagnostic values of other genes were also performed as follows in SONFH samples: LILRB2 (AUC: 0.890), IRF8(AUC:0.883), TYROBP(AUC:0.883), CCR2(AUC:0.867), FPR2 (AUC: 0.850), MNDA (AUC:0.850), TLR8 (AUC:0.850), TLR1(AUC:0.847), TLR2 (AUC:0.843), CCR1(AUC:0.770). Overall, the above 11 hub genes had good diagnostic performance in SONFH, which might be potential biomarkers for SONFH based on our present samples.

### Identification of the tissues

**Fig. 9:**
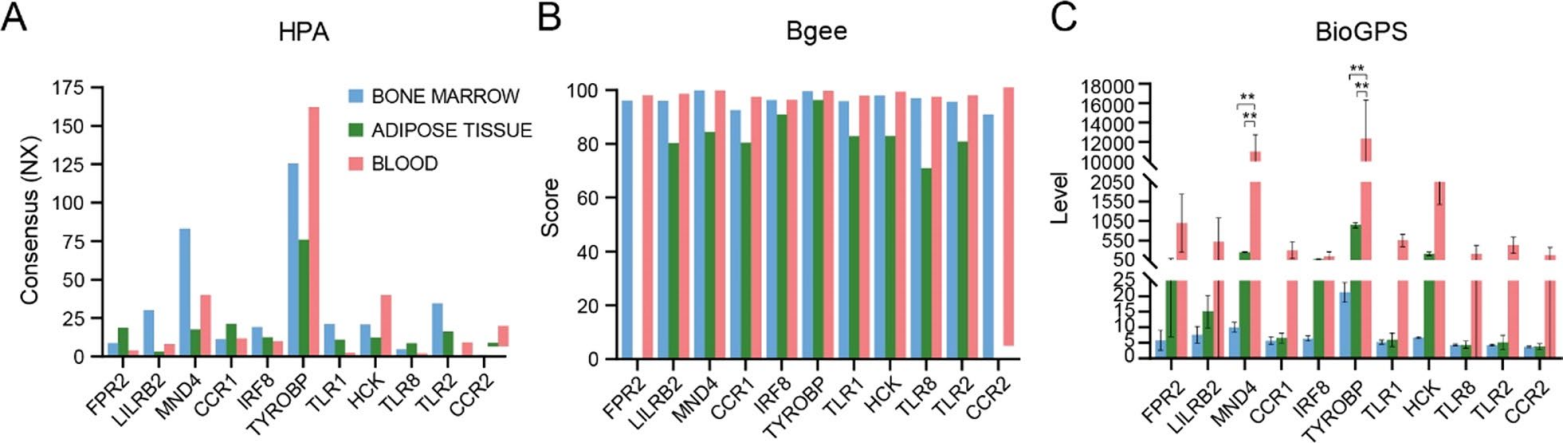
The expression level of hub genes on mRNA in human bone marrow, adipose tissues and blood using by HPA, Bgee and BioGPS. Blue color was bone marrow, and green color was adipose tissue, and red color was blood. ** represents *P* < 0.01

WE analyzed the specific expression level of the 11 hub genes in human bone marrow, adipose tissues and blood using the HPA, Bgee and BioGPS databases. The results of HPA showed that LILRB2, MNDA, TLR1and TLR2 were highly expressed in bone marrow than adipose tissue and blood, FPR2, CCR1 and TLR8 were highly expressed in adipose tissue than bone marrow and blood, and TYROBP, HCK and CCR2 were highly expressed in blood than bone marrow and adipose tissue in Fig. 9A. The specific expression level of all 11 hub genes was of highest available score in blood compared to bone marrow and adipose tissue in the Bgee and BioGPS databases, and FPR2 and CCR2 had no expression score in the Bgee database in Fig. 9B, C. The most highly tissue-specific expression gene was TYROBP in all three databases, and the secondary one was MNDA.

### Construction of the target gene-miRNA network

**Fig. 10:**
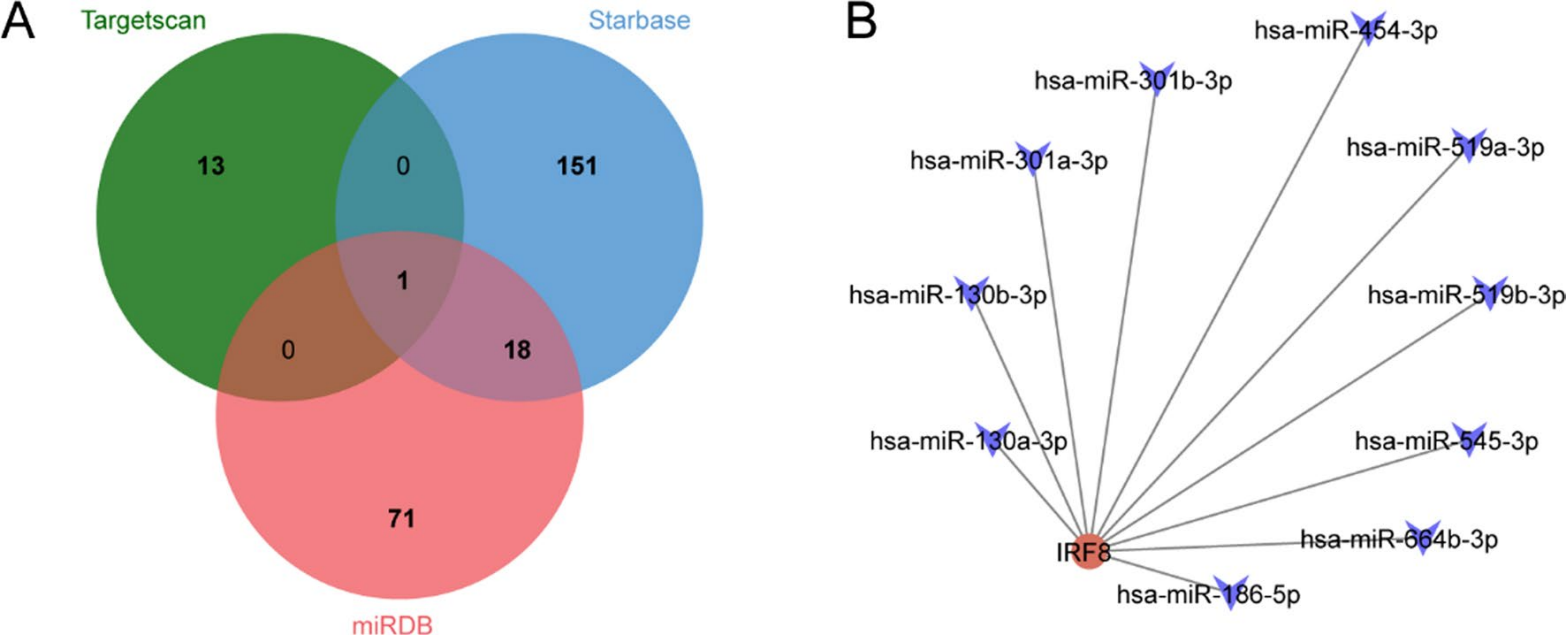
A mRNA-miRNA co-expressed network. **A** Venn diagram of target miRNAs predicted by the hub genes at least 3 datasets. **B** The mRNA-miRNA co-expressed network was constructed by Cytoscape including 11 nodes and 10 edges. Node represented mRNA or miRNA, while edge represented interaction of mRNA and miRNA. Red circles represent the hub genes, and blue V-shapes represent miRNAs

The miRNAs were well known to induce gene silencing and downregulate gene expression by binding to the 3′UTR of target mRNAs. To predict target miRNAs of hub genes, three online miRNA-related database was utilized including Targetsan, miRDB and ENCORI. Finally, 10 mRNA-miRNA pairs that were predicted at least 3 databases, were constructed by Cytoscape. As shown in Fig. 10, only one mRNA, known as IRF8, was retained, which was modulated by 10 miRNAs.

### Prediction of corresponding upstream lncRNAs of hub genes and construction of ceRNA networks

**Fig. 11:**
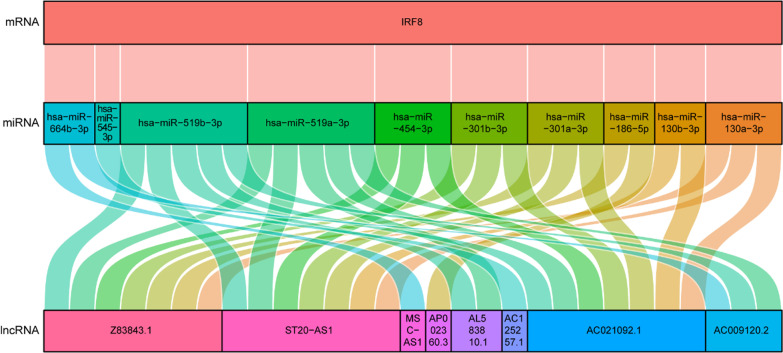
The Sankey diagram of ceRNA networks of IRF8. Upper side of the plots represent the hub genes, and middle side of the plots represent the target miRNAs, and lower side of the plots and yellow diamonds represent the upstream lncRNAs

To reveal the global regulatory network of hub genes, and long noncoding RNAs (lncRNAs) in SONFH, the online database ENCORI 3.0 was used to predict the lncRNAs that interact with the IRF8 and 10 target miRNAs. Finally, we obtained 8 corresponding upstream lncRNAs and 10 target miRNAs and IRF8. The ceRNA regulatory network was constructed and visualized using “ggalluvial” R package in Fig. 11.

### Validation of the hub genes

**Fig. 12:**
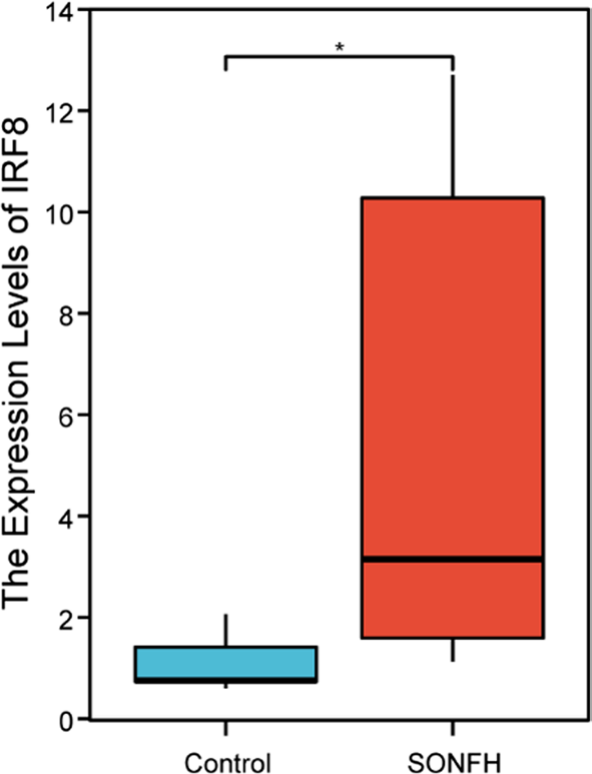
Validation of the expression of IRF8 via qRT-PCR. *P < 0.05

The expression levels of the IRF8 were examined by the qRT-PCR in 14 cartilage samples. IRF8 was reported to be significantly dysregulated in SONFH compared with healthy samples, indicating that the results were reproducible and reliable, as shown in Fig. 12.

### Identification of the potential drugs

**Fig. 13:**
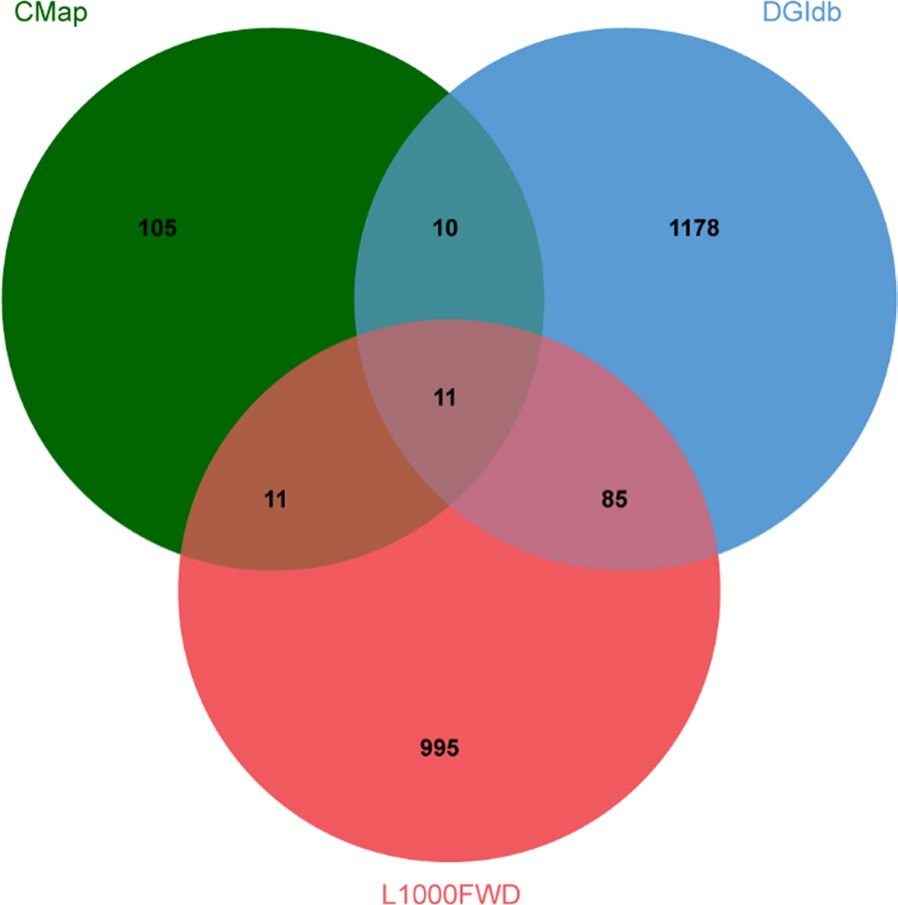
Venn diagram of potential drugs or molecular compounds in the L1000FWD, DGIdb and CMap databases

Using the L1000FWD, 2298 drugs with a similarity score < − 0.03 were screened by a reverse screen of the 372 DEGs in the database. We then analyzed and found 1284 drugs or molecular compounds that could reverse the expression of the 372 DEGs using the DGIdb database. Furthermore, 760 small molecules that could be associated with development of SONFH were identified by the CMap database using the 372 DEGs based on an average score < − 0.65. As shown in Fig. 13 and Table 4, there were 11 potentially overlapping drugs or molecular compounds among L1000FWD, DGIdb and CMap databases as follows: sirolimus, lovastatin, myricetin, rosiglitazone, vinblastine, genistein, estradiol, domperidone, phenformin, vorinostat and fenbufen.

**Table 4:** The connectivity scores and of description 11 potentially overlapping drugs by L1000FWD, DGIdb and CMap databases

Name	Dose	Cell	Score	Mean	Enrichment	*P*	Specificity	Description
Vorinostat	10 µM	HL60	− 0.957	− 0.218	− 0.374	–	–	HDAC inhibitor
Estradiol	10 nM	MCF7	− 0.852	− 0.089	− 0.183	–	–	Contraceptive agent
Fenbufen	16 µM	MCF7	− 0.794	0.079	0.195	0.941	0.9	Cyclooxygenase inhibitor
Phenformin	17 µM	MCF7	− 0.727	− 0.062	− 0.244	–	–	AMPK activator
Rosiglitazone	10 µM	MCF7	− 0.705	− 0.106	− 0.295	0.142	0.2733	Insulin sensitizer
Genistein	10 µM	MCF7	− 0.699	− 0.207	− 0.484	–	–	Tyrosine kinase inhibitor
Domperidone	7 µM	PC3	− 0.69	− 0.068	0.242	–	–	Dopamine receptor antagonist
Vinblastine	100 nM	PC3	− 0.678	− 0.291	− 0.599	0.143	0.3741	Microtubule inhibitor
Myricetin	13 µM	MCF7	− 0.657	− 0.249	− 0.474	0.236	0.2432	Androgen receptor agonist
Lovastatin	10 µM	MCF7	− 0.653	− 0.137	− 0.304	0.757	0.8372	HMGCR inhibitor
Sirolimus	100 nM	MCF7	− 0.651	0.257	0.31	0.000	0.3072	MTOR inhibitor

### Molecular docking between small molecular compounds and the hub genes

**Fig. 14:**
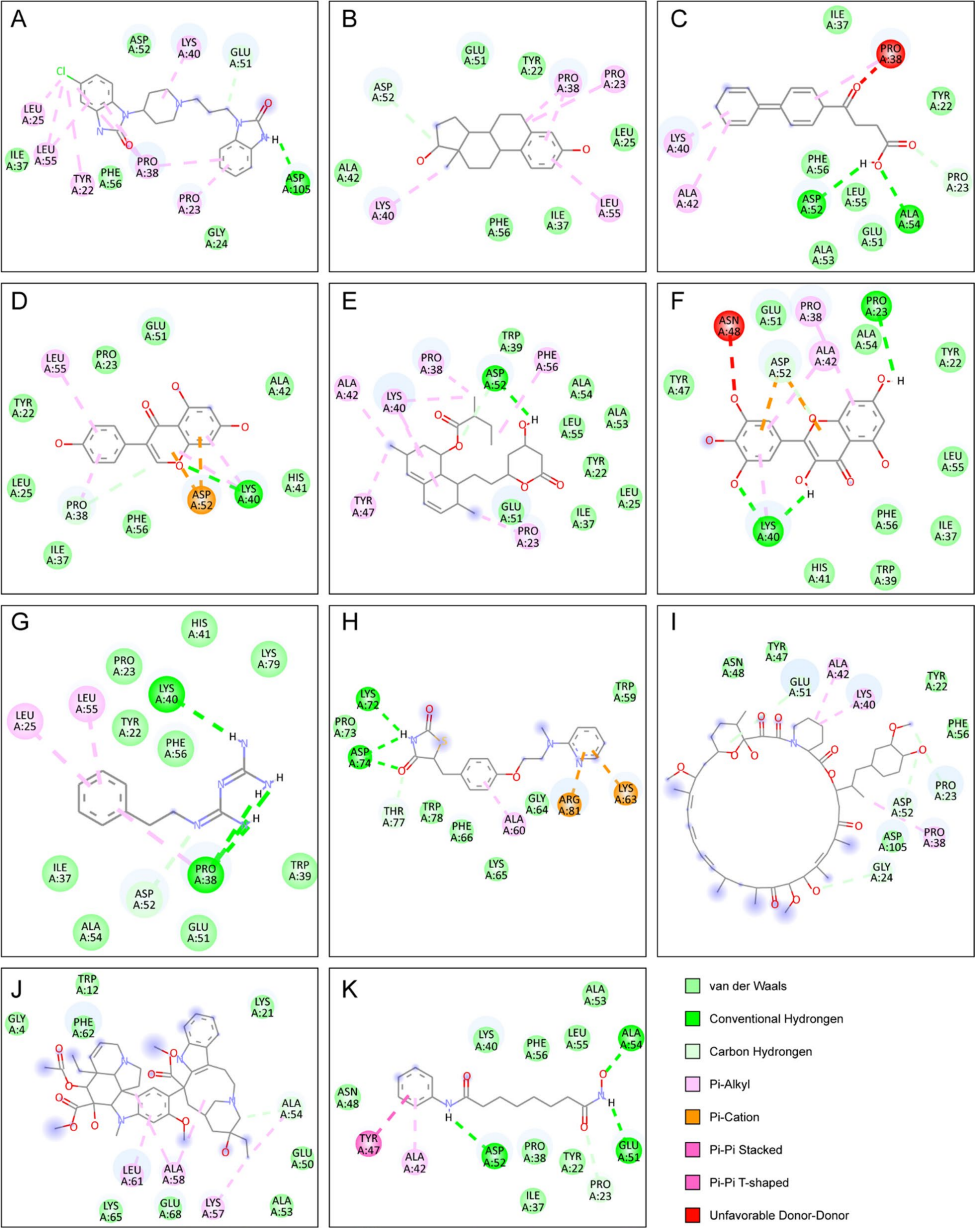
Molecular docking patterns of IRF8 and 11 small molecules complex (**A**–**K**) the 2D molecular docking pattern of IRF8 with vorinostat, estradiol, fenbufen, phenformin, rosiglitazone, genistein, domperidone, vinblastine, myricetin, lovastatin and sirolimus, respectively

There were only IRF8 that was identified by CytoHubba and involved in the ceRNA regulatory network and 11 therapeutic small molecules including sirolimus, lovastatin, myricetin, rosiglitazone, vinblastine, genistein, estradiol, domperidone, phenformin, vorinostat and fenbufen in the intersection of CMap, DGIdb and L1000FWD databases. We carried out molecular docking analysis of 11 small molecules and IRF8. The smaller the binding free energy, the more stable the conformation formed by the ligand and the receptor. The results of binding energy between core target proteins and the key active ingredients are shown in Table 5. Among all the values, the IRF8-estradiol and IRF8-genistein complex had the best binding energy value of − 7.9 kcal/mol, followed by IRF8-domperidone (− 7.8 kcal/mol) and IRF8-lovastatin (− 7.8 kcal/mol) and IRF8-myricetin (-7.8 kcal/mol). The docking models of above IRF8 and their corresponding active small molecules complex having best binding energy values are shown in Fig. 14 and Table 5.

**Table 5:** The total-score of molecular docking of 11 small molecules and IRF8

Ligand	Target protein_PDB ID (kcal/mol)
	IRF8_2dll
Estradiol	− 7.9
Genistein	− 7.9
Domperidone	− 7.8
Lovastatin	− 7.8
Myricetin	− 7.8
Fenbufen	− 7.7
Rosiglitazone	− 7.3
Sirolimus	− 7.2
Phenformin	− 7.1
Vorinostat	− 6.6
Vinblastine	− 6.2

## Discussion

SONFH referred to the series of pathological changes or interruption under the application of glucocorticoid, to the placement of bone marrow components and bone cell death, and subsequent autologous repair leaded to structural changes in femoral head structure and clinical manifestations until collapse [26]. Despite the numerous studies on SONFH, its pathology, pathogenesis and efficacy remain unclear [27]. Once the necrosis of the femoral head occurred, most of the outcome of the lesion progression would be the collapse of the femoral head to different degrees, which would affect the hip function, and some patients would require surgical techniques to prevent or delay the collapse of the femoral head and secondary hip degeneration [28], such as core decompression [29], core decompression combined with bone marrow-derived cell therapies [30] or osteotomy [31], and even eventually undergo artificial joint replacement [5]. At present, there was still no completely satisfactory way to treat SONFH, and early diagnosis, early reasonable and effective treatment might be the best way to preserve the patient's own hip as possible at the current technological level. The ideal state should be treated before the collapse of the femoral head, and early treatment must require an early diagnosis, otherwise SONFH patients would lose the optimal treatment opportunity, resulting in a poor prognosis. Current magnetic resonance imaging (MRI) was the gold standard of early SONFH, and compared to X-ray ray and CT scan technology, MRI could be diagnosed several weeks after patient onset [32]. However, with the understanding of other diseases of the hip joint, such as transient osteoporosis of hip, or bone marrow edema syndrome (BMES), bone cyst of the femoral head, subchondral incomplete fracture and rapid progressive osteoarthritis, SONFH was frequently confused by the above diseases in MRI [33]. Consequently, searching a circulating diagnostic marker with more effective, reliable and high sensitivity and specificity in the early stage of SONFH remained a major clinical challenge. With the rapid development of deep learning technologies including areas of molecular docking, transcriptomics, reaction mechanism elucidation and molecular energy prediction, high-throughput omics provided strong support for the screening of molecular markers and drug prediction and molecular network construction [34]. At the same time, an increasing number of studies showed that the immune cell infiltration played an important role in the development of SONFH and that CIBERSORT tools would contribute to the analysis of immune cell infiltration patterns in the disease [35]. In this study, we would try to optimize the bioinformatics comprehensive analysis parameters to screen potential biomarkers for early diagnosis of SONFH and combine with computer-aided molecular docking technology to predict the binding of potential therapeutic drugs and potential biomarkers and further explore the role of immune cell infiltration in SONFH, which would provide new ways for early diagnosis and drug prediction of SONFH.

We selected and downloaded the GSE123568 dataset from the GEO database and identified a total of 372 DEGs including 175 upregulated genes and 197 downregulated genes in SONFH samples compared to non-SONFH (following steroid administration) Samples based on the defined criteria of an adjusted *P* value < 0.01 and |log_2_FC|> 1. The pathway enriched by GSEA of all genes mainly involved regulation of cytoskeleton organization, nucleobase-containing compound catabolic process, positive regulation of cellular component biogenesis and leukocyte differentiation., which provided some evidence that the most significant enriched gene sets that were correlated with SONFH patient were related to metabolism. In addition, the GO and KEGG enrichment analysis of DEGs were mainly related toneutrophil activation, neutrophil-mediated immunity, neutrophil degranulation, neutrophil activation involved in immune response, cytokine secretion, myeloid cell differentiation, positive regulation of cytokine production, osteoclast differentiation and viral protein interaction with cytokine and cytokine receptor. Cytokines were bioactive small glycoproteins, which could act as cell-to-cell signaling molecules that mediate interactions between immune cells and participate in the inflammatory response [36]. It is well known that cytokines played important roles in cell differentiation, proliferation and immune regulation and binded to cell membrane surface receptors to activate intracellular signaling pathways [37]. Signaling transduction between cytokines and specific cell subsets was essential for maintaining tissue homeostasis in vivo [38]. The above results in GO, KEGG and GSEA enrichment analysis all suggested that the immune activation and signal transduction might play a significant role in SONFH. Yuan et al. had found that the cytokines such as IL-10, IL-12 and TNF-α were implicated in the development of SONFH [39]. The above results were consistent with our analytical data, suggesting that the analytical results of this study were theoretically accurate.

We performed a comprehensive evaluation of SONFH immune infiltration with the CIBERSORT software to further explore the role of immune cell infiltration in SONFH. We found that activated T cells CD4 memory, T cells gamma delta, T cells CD8, eosinophils, B cells naïve, B cells memory, neutrophils, activated NK cells and resting mast cells might be related to the occurrence and development of SONFH. Bradley and his colleagues’ studies showed that the inflammatory infiltrate in SONFH patients comprised primarily CD4(+) T cells and CD68(+) macrophages, the latter of which expressed increased levels of cellular adhesion molecules [40]. Tianbo et al. suggested that polymorphisms of Interleukin-4 (IL-4) gene might be associated with susceptibility to SONFH, which helped reduce the infiltration of M1 phenotypic macrophages and maintain the activation of M2 phenotypic macrophages [41]. Mayu et al. found that neutrophil extracellular traps-forming neutrophils were present in small vessels surrounding the femoral head of patients with ONFH but not osteoarthritis [42]. Zou et al. explained that Th17 cells were recruited to an inflamed synovium, and inflammatory cytokine IL-17 was expressed at an increased level in the hip synovium of ONFH patients, which possibly contributed to clinical syndrome development [43]. Delaere et al. indicated intertrabecular spaces were invaded by connectivo-vascular tissue with focal accumulation of mast cells, and several resorption foci were filled with mononucleated cells in ONFH patient [44]. However, there was no research conducted on specific mechanisms of these immune cells’ correlations in SONFH, and further experimental evidence was required.

Thereafter, the PPI network of these DEGs in SONFH was constructed by using the STRING database, and the node topological features and internode interactions were analyzed to reveal the biological mechanisms associated with the PPI network by using the NetworkAnalyzer plug-in in Cytoscape. The biological networks of the organism with stable balance might be composed of several functional modules, whose complex modules and interactions often lead to the same biological processes, and which provided a new idea for the biological functions that constitute the various components of the complex network. A total of 3 cluster modules from the PPI network were extracted by MCODE analysis based on k-core > 5. Cluster 1, which comprised 19 nodes and 88 edges, showed the highest cluster score (K-core: 9.778); this cluster was all upregulated DEGs including PINK1, BCL2L1, BNIP3L, MAP1LC3B, GABARAPL2, CASP1, PTEN, FAS, BID and FOXO3. The final filtered hub nodes also varied depending on the filtering criteria. These were 11 hub genes involved in SONFH were further identified by intersecting the results within at least three algorithms from the four algorithms of cytohubba including MCC, MNC, DMNC and degree; these genes included FPR2, LILRB2, MNDA, CCR1, IRF8, TYROBP, TLR1, HCK, TLR8, TLR2 and CCR2. All of these hub genes identified from CytoHubba were present within the 3 cluster module results by MCODE in Cytoscape. Eleven hub genes were all upregulated in the SONFH patients, and ROC curve analysis suggested that these genes had higher diagnostic value in combined with their expression levels in the SONFH and non-SONFH samples. Overall, these eleven hub genes played a crucial role in the molecular level of immune response-regulating signaling pathway, inflammatory response, activation of macrophage, cellular response to cytokine stimulus and phagocytosis. The specific expression level of all 11 hub genes was of highest available score in blood compared to bone marrow and adipose tissue in the Bgee and BioGPS databases which provided us with some novel potential therapeutic targets for SONFH.

In addition, we constructed an mRNA-miRNA co-expression network and ceRNA networks to elucidate the pathogenesis of SONFH at the transcriptome level. In this study, only one hub gene in the miRNA-gene network, which were predicted at least Targetsan, miRDB and ENCORI databases, was IRF8. Therefore, we speculated that IRF8 might play an important role in the occurrence and progression of SONFH. Evidence from previous studies in animals and humans showed that IRF8, a transcription factor important for myelopoiesis and osteoclastogenesis, established cell type-specific enhancer landscapes in osteoclast precursors and mature osteoclasts in the orthopaedic disease [45]. We speculated that IRF8 had the potential to be used as a diagnostic marker of SONFH, which had been confirmed to be significant differences between the SONFH and non-SONFH patients in this study, but numerous clinical studies were still needed to verify the diagnostic value of IRF8.

MiRNAs was an endogenous noncoding RNA molecule of length 20-24nt, which could regulate gene expression and repress translation at the post-transcriptional level of target genes by targeting the 3′UTR region of the mRNA [46, 47]. However, the expression level of miRNAs also infected by its upstream molecular lncRNA, which was another noncoding RNA molecule of a transcript greater than 200 bp in length that were no or little translational potential [48]. LncRNAs act as miRNA sponge, contained miRNA response elements and could bind to miRNA, which would prevent miRNA from inhibiting target genes and upregulate corresponding gene expression [49, 50]. Due to their essential role in cell information regulation, noncoding RNA molecule served as a circulating disease diagnostic marker, which received widespread attention as new molecular markers [51]. In our study, we observed all 10 miRNAs that target IRF8, including hsa-miR-130a-3p, hsa-miR-130b-3p, hsa-miR-301a-3p, hsa-miR-301b-3p, hsa-miR-454-3p, hsa-miR-519a-3p, hsa-miR-519b-3p, hsa-miR-545-3p, hsa-miR-664b-3p and hsa-miR-186-5p. Genfa Du showed that hsa-miR-130b-3p as hub miRNA play important roles in adipogenesis and osteogenesis of human BMSCs [52]. Ning Han pointed out that the ceRNAs of lncRNA H19-hsa-miR-519b-3p/hsa-miR-296-5p-ANKH might play important roles in ONFH development based on bioinformatics analysis. Hui Ren suggested that hsa-miR-186-5p as upregulated miRNA might play an important role in glucocorticoid-induced osteoporosis by targeting mRNA and regulating biological processes and bone metabolism-related pathways [53]. At the same time, we predicted 8 corresponding upstream lncRNAs among these selected 10 miRNAs, which included Z83843.1, ST20-AS1, MSC-AS1, AP002360.3, AL583810.1, AC125257.1, AC021092.1 and AC009120.2, andwhich had been confirmed to form CeRNA regulatory relationships with IRF8 in the ENCORI database. Naidong Zhang explored that MSC-AS1 might promote the osteogenic differentiation of BMSCs through sponging microRNA-140-5p to upregulate BMP2 [54]. Zhenyu Tang suggested that MSC-AS1 might regulate the miR-369-3p/JAK2/STAT3 signaling pathway to accelerate the viability and inhibit inflammation and cell apoptosis in OA chondrocytes [55]. Hence, our results laid a foundation for further researches.

To predict the potential effective therapy for SONFH, we applied the CMap, DGIdb and L1000FWD databases to determine 11 therapeutic small molecular compounds that might reverse the abnormally high expression of the 372 SONFH-related DEGs, including vorinostat (HDAC inhibitor), estradiol (contraceptive agent), fenbufen (cyclooxygenase inhibitor), phenformin (AMPK activator), rosiglitazone (insulin sensitizer), genistein (tyrosine kinase inhibitor), domperidone (dopamine receptor antagonist), vinblastine (microtubule inhibitor), myricetin (androgen receptor agonist), lovastatin (HMGCR inhibitor) and sirolimus (MTOR inhibitor). Mounting epidemiological studies confirmed that using these several databases, such as CMap, DGIdb and L1000FWD, was a good strategy and method for conducting drug repositioning research [25]. Xu et al. showed that vorinostat, known as a potent anti-myeloma drug, stimulated the osteogenic differentiation of mesenchymal stem cells in vitro [56, 57]. Fenbufen is a phenylalkanoic acid derivative with analgesic and anti-inflammatory activity, which using in rheumatic diseases and acute pain [58]. Lee et al. found that rosiglitazone coordinated a structural and metabolic remodeling in adipocytes from both visceral and subcutaneous depots that enhanced oxidative capacity [59]. Thangavel et al. summarized the potential benefits of genistein on menopause symptoms and menopause-related diseases like cardiovascular, osteoporosis, obesity, diabetes, anxiety, depression and breast cancer [60]. Imran et al. explored that health benefits of myricetin are related to its impact on different cell processes, such as apoptosis, glycolysis, cell cycle, energy balance, lipid level, serum protein concentrations and osteoclastogenesis [61]. Shahrezaee et al. concluded that the lovastatin and simvastatin efficiently ameliorated the ovariectomized-induced osteoporosis, which affected bone turnover by increasing the osteoblastic bone formation and blocking the osteoclastogenesis [62]. To further confirm our findings, we performed relevant molecular docking on IRF8 with above 11 small molecules. The results showed that all of them could successfully bind to IRF8, which provided more potential options for the treatment of SONFH patients. Although the expression of IRF8 had been validated in both blood and cartilage, and some previous results were consistent with our analysis, the reliability of this study required further experimental validation of the molecular mechanisms.

Although the above approaches elucidate an association between IRF8 and the SONFH and explore the "multi-targets and multi-pathway" patterns in the development and progression of SONFH, our approach still has some shortcomings, such as the reliance on the reliability of the GEO database and variable data quality, which can substantially affect the analysis of gene expression in the results. Further experimental and clinical research on drug efficacy in vitro cell with different level of IRF8 expression and these related noncoding and potential therapeutic molecules is urgently needed to demonstrate their functions.

## Conclusions

In this present study, a comprehensive bioinformatics approach based on the GSE123568-assisted analysis of expression changes in key genes revealed that in SONFH patients compared to non-SONFH patients, we found that 372 DEG co-overlapping, including 197 downregulated DEG and 175 upregulated DEG and that activated T cells CD4 memory, B cells naïve, B cells memory, T cells CD8 and T cells gamma delta might be involved in the occurrence and development of SONFH. At the same time, we identified IRF8,10 target miRNAs, 8 corresponding upstream lncRNAs and 11 potentially drugs, which provided insight into the pathogenesis and treatment of SONFH.

## Data Availability

Publicly available datasets were analyzed in this study. These data can be found at the following URL: GSE123568 dataset (https://www.ncbi.nlm.nih.gov/geo/query/acc.cgi?acc=GSE123568).

## Abbreviations

AUC: Area under the curve
BP: Biological process
BMES: Bone marrow edema syndrome
CC: Cellular component
ceRNA: Competing endogenous RNA
CMap: Connectivity Map
DEG: Differentially expressed gene
DGIdb: Drug Gene Interaction Database
ENCORI: Encyclopedia of RNA Interactomes
FDR: False discovery rate
FC: Fold change
GEO: Gene expression omnibus
GSEA: Gene set enrichment analysis
GO: Gene ontology
HPA: Human Protein Atlas project
KEGG: Kyoto Encyclopedia of Genes and Genomes
MCC: Maximal clique centrality
MNC: Maximum neighborhood component
MCODE: Molecular complex detection
MF: Molecular function
MREs: Mirna response elements
NCBI: National Center for Biotechnology Information
NES: Normalized Enrichment Score
NFH: Necrotic femur head
L1000FWD: L1000 fireworks display
ONFH: Osteonecrosis of the femoral head
PCA: Principal component analysis
PDB: Rotein Data Bank
PPI: Protein–protein interaction
RMA: Robust multiarray average
SONFH: Steroid-induced osteonecrosis of the femoral head
STRING: Search tool for the retrieval of interacting genes
TCM: Traditional Chinese medicine
